# Dysfunction of G Protein-Coupled Receptor Kinases in Alzheimer's Disease

**DOI:** 10.1100/tsw.2010.154

**Published:** 2010-08-17

**Authors:** William Z. Suo, Longxuan Li

**Affiliations:** ^1^ Laboratory for Alzheimer's Disease and Aging Research, VA Medical Center, Kansas City, MO, USA; ^2^ Department of Neurology, University of Kansas Medical Center, Kansas City, KS, USA; ^3^ Department of Molecular and Integrative Physiology, University of Kansas Medical Center, Kansas City, KS, USA

**Keywords:** G protein-coupled receptor kinase (GRK), receptor desensitization, aging, Alzheimer's disease, cholinergic, muscarinic, transgenic mice, β-amyloid

## Abstract

Although mutations and variations of several genes have been identified to be involved in Alzheimer's disease (AD), the efforts towards understanding the pathogenic mechanisms of the disease still have a long journey to go. One such effort is to identify the signal transduction deficits, for which previous studies have suggested that the central problems appear to be at the interface between G proteins and their coupled receptors. G protein-coupled receptor kinases (GRKs) are a small family of serine/threonine protein kinases primarily acting at the “receptor-G protein interface”. Recent studies have indicated the possible involvement of GRK, primarily GRK2 and GRK5, dysfunction in the pathogenesis of AD. It seems that mild, soluble, β-amyloid accumulation can lead to a reduced membrane (functional) and an elevated cytosolic GRK2/5. The increased cytosolic GRK2 appears to be colocalized with damaged mitochondria and neurofibrillary tangles. Moreover, the total levels of GRK2, not only in the brain, but also in peripheral blood samples, are increased in a manner inversely correlated with the patient's cognitive levels. The deficiency of GRK5, on the other hand, impairs presynaptic M2 autoreceptor desensitization, which leads to a reduced acetylcholine release, axonal/synaptic degenerative changes, and associated amnestic, mild cognitive impairment. It also promotes an evil cycle to further increase Beta-amyloid accumulation and exaggerates brain inflammation, possibly even the basal forebrain cholinergic degeneration. Therefore, continuous efforts in this direction are necessary before translating the knowledge to any therapeutic strategies.

## INTRODUCTION

Alzheimer's disease (AD) is one of the most persistent and devastating dementing disorders that affects millions of Americans, but has little or no effective therapies. The patient population keeps growing and is projected to double by 2020. The cost of care for this particular patient population is disproportionately high since they require expensive support. Therefore, advances in the understanding of the disease pathogenesis that can shed light on the improvement of the disease prophylaxis, therapies, and management are highly appreciable and desperately needed.

AD is a neurodegenerative disorder, clinically featured with progressive loss of memory and other cognitive functions, and pathologically featured with accumulation of senile plaques (SPs) and neurofibrillary tangles (NFTs) in association cortices[1,2,3,4]. Although a full picture of the pathogenic processes of the disease remains to be puzzled together, the identification of mutations in β-amyloid (Aβ) precursor protein (βAPP), presenilin 1 and 2 responsible for early-onset familial AD, and apolipoprotein E polymorphisms associated with late-onset or so-called sporadic AD have led to an explosion of the knowledge regarding the disease pathogenesis. Mountains of information in AD research have been reviewed from a variety of perspectives. This mini-review will briefly summarize recent studies regarding the dysfunction of G protein-coupled receptor (GPCR) kinases (GRKs) in AD, and will discuss their relation to known aspects of the disease pathogenesis and future perspectives.

## SIGNAL TRANSDUCTION DEFICITS IN AD CONVERGE TO THE RECEPTOR-G PROTEIN INTERFACE

Cellular health depends on a balance of the cellular signal transduction system. Imbalance of the neuronal signal transduction system is a pathological feature in AD. It displays as (1) increased activity for many protein kinases and/or decreased activity for protein phosphatases, which may contribute to neurofibrillary degeneration[5]; (2) increased proteolytic production of Aβ and/or decreased clearance of Aβ, which result in cerebral β-amyloidosis[6]; and (3) exaggerated activation of microglia and astrocytes, which can lead to inflammatory neuronal damage[7].

Previous studies have found that the signal transduction system in the AD brain is featured with hyperactivity[8], particularly related to various GPCRs and their downstream signaling[8,9,10,11,12,13]. More specifically, several authors have pointed out that the locus of the signal transduction deficits appears to be at the "receptor-G protein interface"[10,14], where GRKs primarily act[15].

## GRKS PRIMARILY ACT AT THE RECEPTOR-G PROTEIN INTERFACE

### GRK Family

GRKs are serine/threonine kinases first discovered through their role in receptor desensitization. GRK family members can be subdivided into three main groups based on sequence homology: rhodopsin kinase or visual GRK subfamily (GRK1 and GRK7), the β-adrenergic receptor kinases subfamily (GRK2 and GRK3), and the GRK4 subfamily (GRK4, GRK5, and GRK6). These kinases share certain characteristics, but are distinct enzymes with specific regulatory properties. GRK2, 3, 5, and 6 are ubiquitously expressed in mammalian tissues, whereas GRK1, 4, and 7 are confined to specific organs. GRK1 and 7 are expressed in retinal rods and cones, respectively, and GRK4 is present in testis, cerebellum, and kidney[16,17,18].

### GRK Function

Structurally, GRKs contain a centrally located 263-266 amino acid (a.a.) catalytic domain flanked by large amino- and carboxyl-terminal regulatory domains. The amino-terminal domains of GRKs share a common size (~185 a.a.) and demonstrate a fair degree of structural homology. These observations have prompted the speculation that amino-terminal domains may perform a common function in all GRKs, potentially that of receptor recognition. The primary function of GRKs is to desensitize activated GPCRs, a negative regulative process, including phosphorylating the activated receptor, uncoupling the receptor-G protein binding, and initiating the receptor internalization. GRKs phosphorylate GPCRs, but only when the receptors are in their activated (agonist occupied) state. The receptor phosphorylation triggers binding of arrestins, which blocks the activation of G proteins, leading to rapid homologous desensitization. As a result of the arrestin binding, phosphorylated receptors are targeted for clathrin-mediated endocytosis, a process that classically serves to resensitize and recycle receptors back to the plasma membrane[19,20,21]. Therefore, the primary function of GRKs is to act at the receptor-G protein interface to desensitize the activated GPCRs.

## Redundancy and Specificity of GRKs

Previous studies, especially *in vitro* studies, have suggested that functional redundancy might exist between the seven isoforms of GRKs in phosphorylating different GPCR substrates; some may even have suggested that there might be little or no specificity[19]. With the generation of GRK transgenic and knockout mice, the functional specificity of GRKs has become clear and convincingly demonstrated *in vivo*. For example, the mice deficient in GRK2, 3, 5, and 6 display selectively impaired desensitization of adrenergic, odorant, muscarinic, and dopaminergic receptors, respectively[22,23,24,25]. The molecular mechanisms underlying this functional specificity *in vivo* remain to be elucidated. However, given the fact that this *in vivo* specificity is often weakened or disappears *in vitro* in cultured cells that artificially overexpress multiple GRK isoforms, it is possible that the functional specificity of GRKs *in vivo* is at least, in part, related to cellular specific expression patterns of the GRK isoforms.

## Subcellular Location of GRKs

GRK2 and GRK5 have been shown to be able to phosphorylate synucleins *in vitro*[26]. The kinase-independent function of GRK has also been reported, such as the suppression of nuclear factor κB (NFκB) activities by GRK5[27,28]. However, there is no doubt that the membrane-integrated GPCRs are still the primary substrates of GRKs. Such a feature determines that GRKs have to be physically associated with the membrane in order to execute their primary function. In resting cells, GRK4 subfamily members (GRK4/5/6) are primarily plasma membrane associated by binding to phosphatidylinositol-4,5-bisphosphate (PIP2) and phosphoserine (PS), and are ready to act when GPCRs are activated by their agonists; while other GRK isoforms are primarily located in cytosol and have to translocate to the membrane in order to function when GPCRs are activated[19,20]. In addition to phosphorylation-dependent kinase activity, many factors regulate GRK activities by altering its subcellular location. For example, PIP2 and $Ca^{2+}$/calmodulin bind to GRK5 at the same site in a mutually exclusive manner to regulate the subcellular location and activity of GRK, with the former keeping it on the membrane and strongly enhancing its activity, while the latter disassociates it from the membrane and strongly inhibits its activity[29,30]. With varied binding sites and affinities, factors like phospholipids (PIP2 and PS) and $G_{\beta\gamma}$ subunits as the membrane binding force, calcium sensor proteins ($Ca^{2+}$/calmodulin and others), caveolin, and stress fibers (F-actin and actinin, perhaps NFTs?) as the cytosolic binding force can more or less affect different GRK isoforms for their subcellular location and activity (Fig. 1)[19,20,21,31,32]. Therefore, it is conceivable that factors that can alter cellular concentrations of those regulatory factors may change the subcellular location and activity of GRKs as well.

## LINKS OF GRK DYSFUNCTION TO AD

The earliest report linking GRK dysfunction to AD was published in 2004 by our group[33]. We were investigating why low-dose, soluble, Aβ-pretreated cells, like microglia or neurons, were hyperactive to

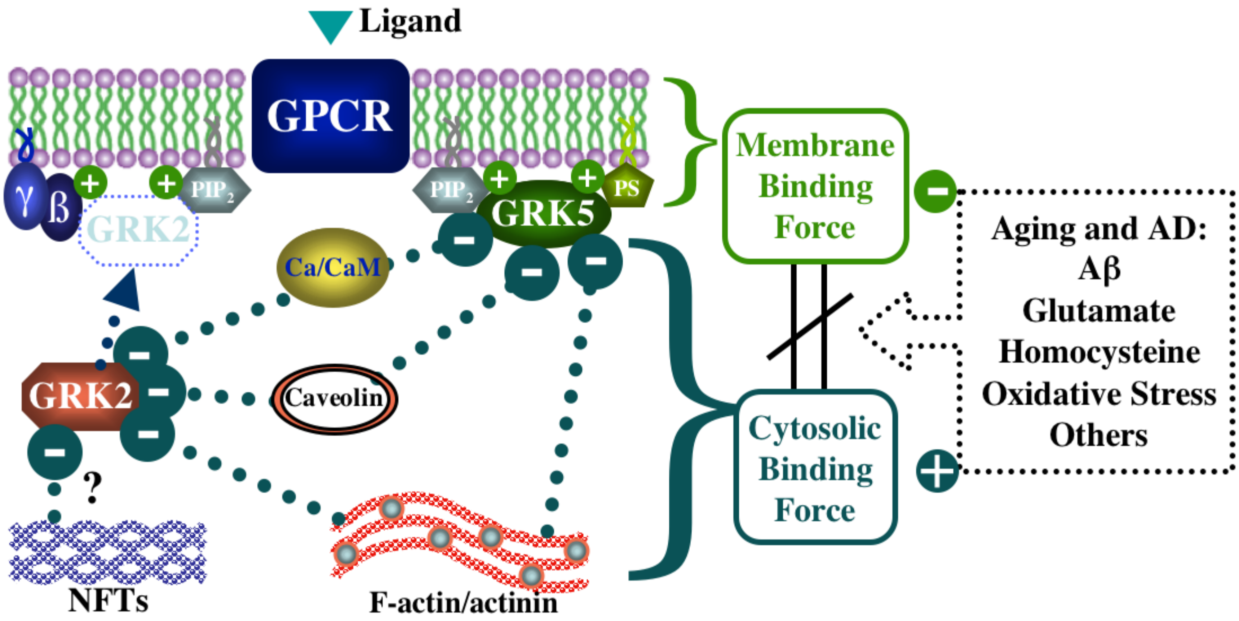

**FIGURE 1.** Regulations of GRK subcellular locations. Balance between membrane (i.e., PIP2 and $G_{\beta\gamma}$ subunits for GRK2, and PIP2 and PS for GRK5) and cytosolic (i.e., calcium sensor proteins, caveolin, and stress fibers) binding forces determines the subcellular locations of GRKs, whereas aging- and AD-related factors may break the balance and push it towards cytosol. Ca/CaM stands for $Ca^{2+}$/camodulin.

certain GPCR stimuli, such as thrombin. We found that the Aβ pretreatment rapidly pushed away membrane-associated GRK5 to cytosol and also prevented GRK2 translocation from cytosol to the membrane after the receptor activation, thus inhibiting the GRK activities. As a result, the thrombin signaling through its GPCR receptors could not be timely desensitized and led to the hyperactivity. Although the mechanisms for how Aβ altered the GRK subcellular location remain to be investigated, it is not hard to imagine that the known effects of Aβ on intracellular calcium concentration and membrane phospholipids may have played significant roles. Beyond the *in vitro* study, we also found *in vivo* evidence in this regard: the membrane content of GRK2 and GRK5 was decreased, while the cytosolic content was increased in the TgCRND8 transgenic mice. Moreover, such changes began at the prodromal stage and lasted until the late stage. The end-stage changes of the GRK subcellular location were later confirmed in postmortem human AD brain samples[34]. It is worth noting that we only looked for changes in GRK2/5, because they are ubiquitously distributed and may have more relevance to our own interests. This does not exclude possible changes in other GRK isoforms, especially considering the broad changes in membrane phospholipids and intracellular calcium in AD or aging brains, which should not only affect GRK2/5, but may influence other GRK isoforms as well.

## GRK2 Dysfunction and AD

As detailed in our initial report[33], the changes of GRK2 in AD transgenic brains include two aspects: the moderate decrease of membrane content and drastically increased cytosolic content. In addition, the total levels of GRK2 in these mice were also significantly up-regulated. Later studies confirmed the GRK2 overexpression in human AD brain samples, and also demonstrated that GRK2 accumulated near damaged mitochaondria and NFTs[35,36]. Interestingly, the total GRK2 levels in peripheral blood samples of AD patients were found not only to be higher than controls, but also inversely correlated to the cognitive levels of AD patients[37]. From the GRK2-deficient mice, we learned that the impact of GRK2 deficiency appears to be limited to the cardiovascular system via its uncompensatable defect in desensitization of β-adrenergic receptors[22,38]. Therefore, if there is any pathologic impact from the moderate decrease in the membrane GRK2 content in AD, it might be limited to an effect through the cerebrovascular system. For potential impact of the increased cytosolic or total GRK2 in AD, one would imagine it could go beyond the cerebrovascular impact, given its ubiquitous distribution and its colocalization to damaged mitochondria and NFTs. Unfortunately, the available GRK2 transgenic mice were made to overexpress GRK2 only in the cardiovascular system[39,40], which did provide the platform to confirm the roles of GRK2 in the cardiovascular system via the β-adrenergic receptors, but were not useful for determining its potential impact on other systems. Therefore, the available evidence so far for GRK2 dysfunction in AD stays at the correlation level, and the efforts to determine the possible value of GRK2 dysfunction in AD pathogenesis are limited to bridging the gap in understanding of the heart-brain connection in relation to neurovisceral damage and vascular complications in AD[41].

### GRK5 Dysfunction and AD

The changes of GRK5 in AD transgenic mouse brains also include the decrease of membrane and increase of cytosolic content, but are not significant for the total level[33]. Similar to the GRK2 transgenic mice, the GRK5 transgenic mice were also created with a cardiac-specific promoter[42] and were inappropriate for studying the impact of GRK5 overexpression on noncardiovascular tissues. Nonetheless, the most recent *in vitro* studies have reported that the RH domain of GRK5 (a.a. 50-176) inhibits NFκB via a kinase-independent mechanism and leads to apoptosis in tumor cells[27,28]. Therefore, should the effect also be true *in vivo*, the increased cytosolic GRK5 may promote neuronal degeneration. For the membrane GRK5 deficiency, on the other hand, the GRK5 knockout (GRK5KO) mice provide a valuable model, and studies using this tool have yielded significant insights into roles of the GRK5 deficiency in AD pathogenesis.

## ROLES OF MEMBRANE GRK5 DEFICIENCY IN AD PATHOGENESIS

GRKs have to be either prelocated at the membrane, such GRK5, or recruited to the membrane to execute their primary function on GPCR desensitization. Especially before the kinase-independent function of GRK5 was identified, the desensitization of GPCRs was thought to be their entire function. Therefore, the term "GRK5 deficiency" in previous literature has been used to refer specifically to the membrane or functional GRK5 deficiency, rather than a down-regulation of total GRK5. In this article, we will continue using this term for the convenience of description.

### Factors Leading to GRK5 Deficiency

As mentioned earlier, the subcellular location of GRK5 is regulated by the balance between the membrane binding force (i.e., phospholipids) and cytosolic binding force (i.e., calcium sensor proteins). The details in this regard, including the specific binding sites and affinities, have been reviewed previously[20,31]. What we want to emphasize here is AD- or aging-related factors that can break the regulatory balance and lead to the membrane GRK5 deficiency. As mentioned, low-dose, soluble Aβ is one of them[33], clearly related to AD. Beyond this, our unpublished data indicate that glutamate, homocysteine, and even oxidative stress can all cause the membrane GRK5 deficiency. In fact, although total GRK5 is up-regulated with increasing age, aged mice still display significant membrane GRK5 deficiency as compared to young ones. These preliminary observations have led us to speculate that the membrane GRK5 (perhaps other isoforms as well) deficiency may be a generic alteration associated with normal aging and is worse in AD (because of the high levels of Aβ and homocysteine, etc.). Therefore, specific efforts in this regard have to be made in the future to validate these thoughts.

## Pathologic Impact of the GRK5 Deficiency

The evidence for this part of our work was obtained primarily from the GRK5-deficient mouse models, such as GRK5KO mice and GRK5 deficient APPsw (Tg2576) double mice (GRK5KO/APPsw). It is worth noting that the GRK5KO mice were created by targeted deletion of exons 6 and 7 of the murine GRK5 gene, and it was predicted that these mice would produce a transcript encoding a.a. 1-178 (which covers the RH domain a.a. 50-176), but exon 9 would be spliced out of frame and would lead to termination after the addition of 16 novel residues[24]. This means that the GRK5KO mice are only deficient in the kinase activity–dependent functional form of GRK5, while the kinase-independent or RH domain–dependent part of the GRK5 remains unaltered. In other words, the phenotypes revealed in these animals are irrelevant to the recently proposed RH domain–dependent function of the GRK5. In addition, compared to other AD mouse models, no genetic modifications were made to those commonly known AD-relevant genes, such as βAPP, presenilins, tau, or apolipoprotein E, etc. Therefore, the phenotypes of these mice are solely caused by the loss or lack of the GRK5 kinase activity.

The initial characterization of the mice revealed that the young mice exhibited mild spontaneous hypothermia as well as pronounced behavioral supersensitivity (i.e., hypothermia, hypoactivity, tremor, salivation, and antinociception) upon challenge with the nonselective muscarinic agonist oxotremorine[24]. Our later characterization in the aged, unchallenged mice discovered that these animals displayed significant short-term working memory deficit, along with hypoalertness[43]. Moreover, this amnestic, mild cognitive impairment (MCI) was more or less correlated to moderate levels of pathologic alterations, including axonal defects (swollen axonal clusters), decreased synaptic proteins (SNAP-25, synaptotagmin, and growth-associated protein-43), and muscarinic receptors (M1 and M2), as well as increased soluble Aβ and tau phosphorylation levels in the hippocampus. Although the synaptophysin level, the standard marker for synaptic degeneration, did not change significantly in the initial study using mixed genders, it was later found that the synaptophysin in the GRK5KO mice was significantly reduced for the female, but not the male, mice[44]. Other pathologic changes were also worsened in the female mice. Beside these changes, there were barely any observable SPs or inflammatory changes, except for a few "micro" plaques captured under the electronic microscope that showed fibrillar Aβ-like structures and degenerating axonal components wrapped by reactive astrocytes. In addition, stepwise-forward discriminant function analysis indicated that different genotype groups (GRK5$^{+/+}$, GRK5$^{+/-}$, and GRK5$^{-/-}$) were apparently distinguishable, which supported that there was a significant GRK5 gene-dose effect. Therefore, it seems that the GRK5 deficiency alone can cause amnestic MCI and promote several AD-related pathologic changes during aging.

In respect to the mechanisms, GRK5 deficiency was initially found to impair desensitization of muscarinic receptors selectively[24]. We recently confirmed the finding and further demonstrated that the selectivity was primarily for M2, partially for M4, but not for the M1 subtype of the muscarinic receptors[45]. It is known that M1, M2, and M4, but not M3 and M5, are enriched in the hippocampus, with M2/M4 being primarily presynaptic autoreceptors to regulate acetylcholine (ACh) release negatively in hippocampal memory circuits[46,47]. Therefore, the potential pathological impact of the GRK5 deficiency may be twofold: (1) it directly causes prolonged presynaptic M2/M4 autoreceptor signaling or *presynaptic cholinergic hyperactivity*; and (2) the presynaptic cholinergic hyperactivity in turn leads to a reduced ACh release in hippocampal memory circuits and results in *postsynaptic cholinergic hypoactivity*, including postsynaptic M1 hypoactivity. In this regard, our data indeed confirmed that the GRK5 deficiency led to a reduced ACh release[45].

The ongoing investigation is still trying to determine whether the impaired M2 desensitization is responsible for most of the pathologic and behavioral changes in the GRK5KO mice. Nevertheless, available evidence appears to support such a possibility. For example, previous studies have suggested that signaling of M1, M3, and M5, but not M2 or M4, appears to be antiapoptotic[48]. M1 signaling has also been shown to inhibit β-amyloidogenic APP processing and to decrease tau phosphorylation *in vitro*[49,50,51]. Moreover, the postsynaptic cholinergic actions also mediate the role of the cholinergic system in cognition[52]. Therefore, the postsynaptic M1 hypoactivity may at least in part contribute to the increased soluble Aβ, tau phosphorylation, and the mild cognitive decline in the GRK5KO mice.

In addition to the inhibited ACh release, would the prolonged/persistent M2 signaling have any impact on the cholinergic neuron itself? It is well established that M1 (M3/M5) and M2 (M4) are typical $G_q$- and $G_i$-coupled receptors, respectively, and often mediate distinct or even opposing signals[53,54]. M2 signaling is known to reduce the cAMP level[53,54] and down-regulate protein kinase A (PKA) activity, a vital signaling pathway for cell survival and apoptotic resistance[55,56,57,58,59,60]. If the M1 signaling is antiapoptotic[48], would the persistent M2 signaling promote apoptotic degeneration of the cholinergic neurons or synapses? The answer for this question may ultimately determine whether the presynaptic M2 hyperactivity is responsible for the cholinergic degenerative changes in the GRK5KO mice.

In addition to the GRK5KO mice, GRK5-deficient APPsw mice were created by cross-breeding the GRK5KO mice and APPsw transgenic mice. Characterization of these double mice is currently still ongoing, but the initial examination of the aged mice found significantly exaggerated inflammatory changes, including microgliosis and astrogliosis, in the brains of the double, as compared to the APPsw mice[61]. The GRK5KO mice showed almost no inflammatory changes, but when crossed with APPsw mice, the double mice showed exaggerated inflammation, much worse than the APPsw mice. This exaggeration is certainly not an additive, but a synergistic effect between the GRK5 deficiency and APPsw overexpression. It is clear that GRK5 itself does not initiate any signaling; rather, it modifies the signaling strength and duration once the signaling is initiated. If the inflammatory signaling is never initiated, such as in the case of GRK5KO mice, the role of GRK5 deficiency may be silenced. In the case of the double mice, however, overexpression of Swedish APP gene produces excess Aβ and its fibril, which may well serve as the inflammatory initiator, and the role of GRK5 deficiency can then be revealed. In AD, fibrillar Aβ itself has been shown to activate a GPCR formyl chemotactic receptor 2 (FPR2 or FPRL-1)[62,63]. Many other intermediate inflammatory responses that are associated with fibrillar Aβ (i.e., C3a, C5a, monocyte chemoattractant protein-1, macrophage inflammatory protein 1, interferon-inducible protein-10, and interleukin-8)[64,65,66,67] are mediated through GPCRs, such as C3aR and C5aR anaphylatoxin receptors[68] and all CCRs, CXCRs, and CXCXRs chemokine receptors[66]. GRKs are known to regulate many of these GPCRs[68,69], including uncompensatable selective regulation. For example, GRK6 has been demonstrated to regulate CXCR4 selectively[70]. Therefore, it is possible that GRK5 may selectively regulate one or more of these GPCR-mediated inflammatory processes, and its deficiency then amplifies the inflammation responses in the double mice. In addition, we have observed a moderate increase in soluble Aβ in the GRK5KO mice[43]. It is possible that the GRK5 deficiency may promote the total Aβ accumulation in the double mice, which then leads to the more severe inflammation (Fig. 2). Therefore, future studies are necessary in order to determine the actual underlying molecular mechanisms before appropriate therapeutic targets can be defined.

## Relation of the GRK5 Deficiency to Existing AD Hypotheses

Debates on detailed causes and processes of AD have been going on for years and will not end soon. Among the existing hypotheses of AD, the cholinergic hypothesis and amyloid hypothesis are two mainstream hypotheses that have been better received and have largely driven the translational and pharmaceutical research of AD in the recent decades[71].

The cholinergic hypothesis states that central cholinergic neuronal dysfunction is largely responsible for the cognitive decline in AD[72]. The amyloid hypothesis proposes that Aβ is the central pathogenic molecule in AD[6]. Although the detailed descriptions may evolve over time with increasing insights into the disease pathogenesis, the principal concepts of these hypotheses appear to stand solidly up to now[6,71,73,74,75,76,77,78]. In fact, rather than being in contrast in any way, these two hypotheses simply focus on different stages and aspects of the disease pathogenesis. For example, the amyloid hypothesis emphasizes the excessive Aβ as the central cause of AD, while the cholinergic hypothesis

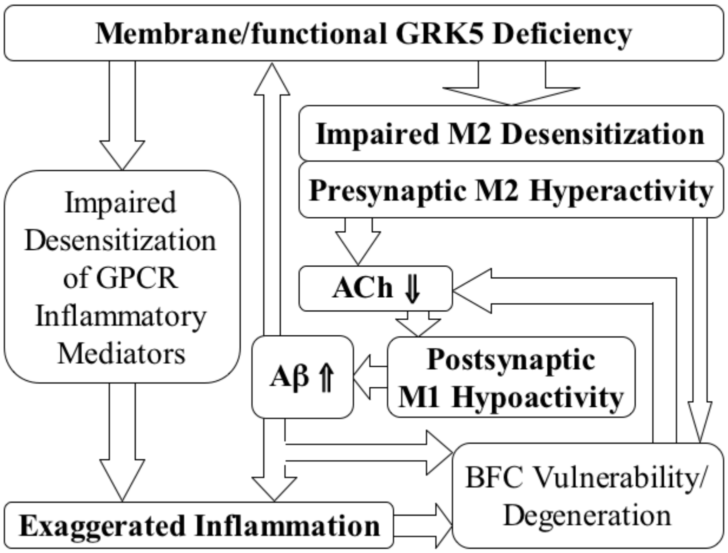

**FIGURE 2.** Schematic illustration of potential pathogenic impact of GRK5 deficiency. The schema is self-explanatory. BFC stands for basal forebrain cholinergic.

accentuates that no matter what initiates the disease process, it has to lead ultimately to the cholinergic dysfunction so to cause the cognitive decline in AD. Therefore, there is a good chance that the two hypotheses may eventually integrate together to better explain the disease process. For the latter, the knowledge gained from studies of the GRK5 deficiency may provide specific links in between.

On one hand, Aβ is one of the main causes for the GRK5 deficiency[33]; on the other hand, the GRK5 deficiency selectively impairs the cholinergic function[45]. Many studies[50,79,80,81,82], including our own[43], have shown that cholinergic dysfunction, especially postsynaptic cholinergic hypoactivity, affects APP processing in favor of the β-amyloidogenic pathway, and therefore further promotes Aβ production. These steps form a vicious circle to accelerate AD pathogenic processes, including both Aβ accumulation and cholinergic dysfunction, while the GRK5 deficiency is a pivotal mediator that links both the amyloid and the cholinergic hypotheses together.

## CONCLUSIONS AND FUTURE PERSPECTIVES

Although studies regarding GRK dysfunction and AD pathogenesis remain to be matured, available evidence indicates that the GRK dysfunction is involved in most of the AD pathogenic processes, from Aβ accumulation, tau phosphorylation, cholinergic hypofunctioning and degeneration, brain inflammation, and cognitive decline, perhaps even cerebrovascular complications as well. From the perspective of GRK5 deficiency, the presynaptic M2 hyperactivity is not only a novel concept, but it also has a strong potential to become an ideal preventive and/or therapeutic target, not only for combating most of the pathologic impact posed by the GRK5 deficiency, but also for breaking the vicious circle between the Aβ and cholinergic dysfunction.

Of course, before translating these researches to the clinical setting, several important questions remain to be addressed. The factors (i.e., Aβ, glutamate, homocysteine, and oxidative stress) that can cause the GRK subcellular location change broadly exist in AD brains and even in normal aging brains to some extent. If they cause the change through altering the balance between the membrane and cytosolic binding forces, it is likely that the resulting GRK subcellular location change is not limited to GRK2 and GRK5. Therefore, we first need to make clear how broad and specific the GRK dysfunction may be in

AD patients. This will help to estimate the overall weight of the GRK dysfunction in the AD pathogenesis. Specifically for each GRK isoform, potential pathologic impact needs to be carefully evaluated and the underlying molecular mechanisms elucidated. For GRK5 deficiency in particular, determination of whether the presynaptic M2 hyperactivity indeed causes cholinergic neuronal or synaptic apoptosis should have a higher priority. Because if the prediction is true, the clinical use of cholinesterase inhibitors, four out of five FDA-approved medications for AD, could have potential adverse effects by worsening the presynaptic M2 hyperactivity, especially when high doses are used. Of course, it needs to be clarified whether the presynaptic cholinergic hyperactivity and postsynaptic cholinergic hypoactivity lead to increased Aβ accumulation and the exaggerated brain inflammation. If not, which inflammatory mediators are under specific regulation by GRK5 and mediate the exaggerated inflammation in the case of GRK5 deficiency? The answers for these questions are essential for defining potential therapeutic targets in the future.

## ACKNOWLEDGMENTS

This work was supported by grants to WZS from the Medical Research and Development Service, Department of Veterans Affairs, the American Federation for Aging Research, and resources from the Midwest Biomedical Research Foundation.
